# Determination of Tetracycline, Oxytetracycline, Sulfadiazine, Norfloxacin, and Enrofloxacin in Swine Manure Using a Coupled Method of On-Line Solid-Phase Extraction with the UHPLC–DAD

**DOI:** 10.3390/antibiotics10111397

**Published:** 2021-11-13

**Authors:** Mohamed S. Gaballah, Xin Li, Zijia Zhang, Abdulaziz Al-Anazi, Hui Sun, Mostafa Sobhi, Mperejekumana Philbert, Mohamed A. Ghorab, Jianbin Guo, Renjie Dong

**Affiliations:** 1 Bioenergy and Environment Science & Technology Laboratory, College of Engineering, China Agricultural University, Beijing 100083, China; saadga22@gmail.com (M.S.G.); lxin@cau.edu.cn (X.L.); zijiazhang@cau.edu.cn (Z.Z.); huisun@cau.edu.cn (H.S.); philbertson2@yahoo.com (M.P.); Dong@cau.edu.cn (R.D.); 2 Department of Marine Environment, National Institute of Oceanography and Fisheries, NIOF, Alexandria 21556, Egypt; 3 Department of Chemical Engineering, College of Engineering, King Saud University (KSU), P.O. Box 800, Riyadh 11421, Saudi Arabia; aalanazi15@ksu.edu.sa; 4 Agricultural and Bio-Systems Engineering Department, Faculty of Agriculture, Alexandria University, Alexandria 21511, Egypt; Msi1433@gmail.com; 5 Office of Chemical Safety and Pollution Prevention, U.S. Environmental Protection Agency (EPA), Washington, DC 20004, USA; ghorab.mohamed@epa.gov; 6 Wildlife Toxicology Laboratory, Department of Animal Science, Institute for Integrative Toxicology (IIT), Michigan State University, East Lansing, MI 48824, USA; 7 Yantai Institute, China Agricultural University, Yantai 264032, China

**Keywords:** UHPLC, on-line SPE, swine manure, veterinary antibiotics

## Abstract

The use of various veterinary antibiotics (VAs) in animal husbandry raises serious concerns about the development of antibiotic resistance. Antibiotics such as tetracycline, oxytetracycline, sulfadiazine, norfloxacin, and enrofloxacin are the most frequently used antimicrobial compounds in animal husbandry and generate large eco-toxicological effects; however, they are still difficult to determine in a complex matrix such as swine manure. This study has developed an effective method for detecting five VAs in swine manure using Ultra-High-Performance Liquid Chromatography–Diode Array Detector (UHPLC–DAD) coupled with on-line solid-phase extraction (SPE). The results show that the mobile phase of ACN/0.01 M oxalic acid was the optimum at pH 3.0. VAs in a swine manure matrix were extracted using solid extraction buffer solution (T3) with 97.36% recovery. Sensitivity, accuracy, and precision were also evaluated. The validity study showed good linearity (R^2^ > 0.99). Limit of detection (LOD) was found to be from 0.1 to 0.42 µg mL^−1^ in the liquid fraction and from 0.032 to 0.58 µg g^−1^ dw in the solid fraction. The corresponding values of the limit of quantification (LOQ) ranged from 0.32 to 1.27 µg mL^−1^ for the liquid fraction and from 0.096 to 1.77 µg g^−1^ dw for the solid fraction. Therefore, the proposed method showed the potential applicability for detecting different antibiotic compounds from swine manure samples.

## 1. Introduction

Veterinary antibiotics (VAs), including tetracyclines (TCs), sulfonamides (Sulfs), and fluoroquinolones (FQs), are frequently used to promote animal growth and prevent disease in the livestock industry [1,2]. Because of their widespread misuse and difficulty to digest in the animal’s gut, a certain amount of VAs is excreted into the animal’s feces [2,3]. Furthermore, the residues of these VAs are toxic and can cause serious allergic reactions in the environment. Animal manure is frequently employed in agriculture, resulting in the introduction of antibiotic resistance genes (ARGs) into the soil system [4]. China is one of the biggest VAs producers worldwide, with tetracycline accounting for half of all VAs produced each year (including antibiotics used in animal feed) [5]. Previous studies have described microbiological and thin-layer chromatography methods for monitoring VAs in biological matrices [6,7,8]; however, most of those studies have focused on one group of VAs only. Several recent studies used HPLC for TCs, sulfonamides, and FQs quantification in other animals’ matrices [9,10,11,12,13].

High-performance liquid chromatography (HPLC) in the reverse-phase mode, with different detection modes, is commonly used to determine VAs. Between them, TCs are frequently investigated; however, they are unstable compounds due to high light sensitivity and the formation of epimers under acidic conditions. TCs in honey were detected using on-line SPE coupled with HPLC by Li et al. [7], and in chicken meat and liver by Shalaby and Yu using HPLC [2]. HPLC was also used to determine FQs in human plasma [14,15] and Sulfs in manure and solid matrices [16]. This implies that TCs, Sulfs, and FQs can be successfully determined using HPLC coupled with different detection modes in various matrices; however, only a few methods have been developed for animal manure samples.

Moreover, most of the procedures involved a complicated extraction step such as SPE, which is time consuming and has poor and inconsistent recoveries [5]. Thus, HPLC coupled with on-line SPE could offer several advantages, including reducing time and chemicals used, in addition to avoiding the common traditional problems associated with off-line SPE [7]. In addition, [17,18] it has been reported that on-line SPE can be used to reach good LOQ levels in several analytical procedures. Therefore, this study aims to establish a simple but effective analytical method for determining five VAs (TC, OTC, SDZ, Norf, and Enorf) from swine manure matrix, depending on an on-line SPE technique coupled with UHPLC–DAD. In addition to this, choosing a suitable mobile phase and different solid extraction solutions were examined to achieve high recovery performance.

## 2. Results

### 2.1. Optimization of the Chromatographic Conditions

#### 2.1.1. Selection of the Optimum Detection Wavelength

UHPLC with UV detectors are the most commonly used techniques for detecting Vas, due to their availability and convenient use in many labs. Diode array detectors (DAD) are more popular in use, since they have a wide UV spectrum (scanning range) covering a wide range of compounds. Here, in this study of the targeted VAs, each antibiotic has a certain wavelength range, for instance, wavelengths of 360 nm for TCs, 270 nm for SAs, and 280 nm for FQs. In this regard, a wide range of wavelengths was examined, including 230, 250, 251, 255, 260, 265, 267, 270, 273, 277, 280, 285, 290, 295, 320, 350, 355, 360, 365, and 370. Finally, 270 and 277 nm were found to be suitable by delivering high peak values for the targeted VAs (Table 1).

#### 2.1.2. Selection of the Suitable Mobile Phase and Chromatographic Conditions

**Figure 1:**
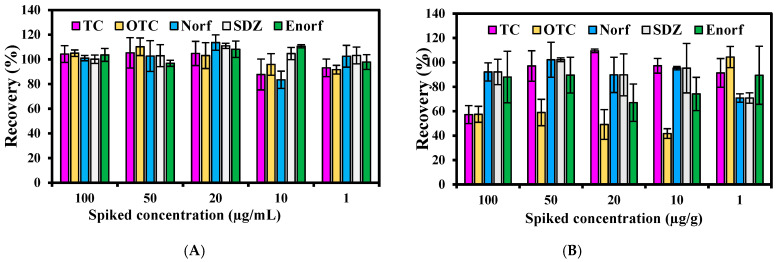
VAs recovery in liquid fraction (**A**) and solid fraction (**B**) at pH 1.5, mobile phase (1).

**Figure 2:**
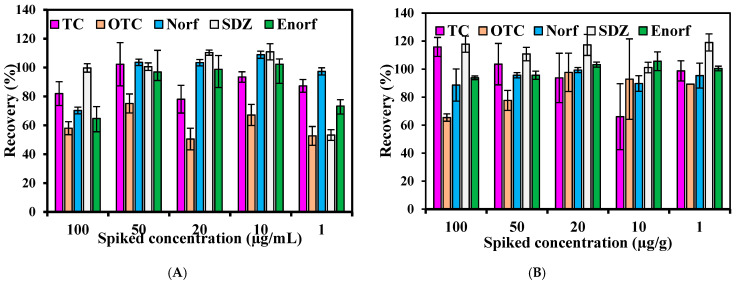
VAs recovery in liquid fraction (**A**) and solid fraction (**B**) at pH 3.0, mobile phase (2).

Generally, in the reversed-phase column, antibiotics might easily show a tailing peak due to metal impurities or other residues [2]. Mobile phase acidity and reversed-phase column properties are responsible for peak quality. In order to avoid the formation of these peaks and adsorption of metal ions on the silanol group in the reversed column, a mobile phase containing various acids has been recommended [7]. Acids in the mobile phase act as a simple ionization suppression agent to minimize mixed separation mechanisms. The acids often used are formic acid and oxalic acid, which are able to sufficiently remove the effect of metal impurities and other residues on the stationary phase. Thus, oxalic acid was chosen in this study due to its ability to be used on wide ranges of VAs groups’ separation through HPLC–UV. The mixture of ACN/oxalic acid/MeOH was previously recommended in the literature due to its ability to provide and improve the peak profiles. A mixture of ACN/oxalic acid was used in this study to separate targeted VAs from swine manure. Mobile phase pH content is of the most interest in light of the detection of analytes using HPLC, which is usually paired with the acid concentration. Thus, two pH levels (1.5 and 3.0) paired with two different concentrations of oxalic acid (0.05 M and 0.01 M) in the mobile phase were accomplished. The detection of targeted VAs under both mobile phases examined in this study is shown in Figure 1 and Figure 2. The recovery of the targeted antibiotics used for comparison and assisting the mobile phase selection was observed, where the recovery of five VAs from both the liquid and solid fractions of swine manure was also observed. On average, 103% of the recovery of VAs was recorded in the liquid fraction, as opposed to 84.4% in the solid fraction with the mobile phase (1) (Figure 1), while an average recovery of 97.4% was obtained in liquid manure using the mobile phase 2 (pH 3.0) against an 86.5% average recovery in the solid fraction (Figure 2). The SDZ recorded the highest average recovery in liquid with both mobile phases, while OTC had the lowest average recovery. Since the difference between both mobile phases is not large, the selection could be due to other findings. At a low pH, the efficiency of the separation of VAs is a little higher; however, this low pH could create many problems for the HPLC machine. When the pH was 1.5, the system’s valves and transition lines were blocked during the operation running, which (although unlikely) could have affected the detection performance with time. Thus, in light of this finding, mobile phase (2) (pH 3.0) is recommended to avoid this issue and keep the machine stable.

Additionally, the ratio between the selected organic solvents, ACN and oxalic acid, was suggested and examined to optimize the mobile phase operation. In this regard, a gradient elution condition was followed, the retention time of five VAs extended, and the peak width satisfied. The mobile phase compositions for both HPLC–UV and on-line SPE are illustrated in Table 2. Thus, good separation was achieved by UHPLC–UV combined with on-line SPE. For UHPLC–UV, a composition of 40:60 ACN/Oxalic acid (0.01 M) was used after many trials. A retention time of 35 min was applied for all antibiotics together in the same run, including 5 min with 100% of MeOH for washing. A 5:95 gradient elution condition was used for on-line SPE until 10 min had elapsed, then switched to 80:20 until 20 min, then returned to 5:95 until 30 min and 5 min with 100% MeOH for washing.

### 2.2. Optimization of SPE

#### 2.2.1. The Choice of On-Line SPE Column Sorbent

SPE is commonly used to clean up samples due to its carbon backbone, aromatic region, and varied functional groups. Direct desorption of analytes from the pre-concentration column to the HPLC column by an optimal eluent for chromatographic separation is important. On-line SPE coupled with HPLC offers a fast and robust method for antibiotics detection. In this study, on-line SPE was applied to clean up the targeted VAs, which represent different classes and have different functional groups that make the detection process difficult. In this regard, to match the sorbent of VAs with analytical column C18, three different extraction solutions in solid fractions were used and evaluated.

#### 2.2.2. Optimal Extraction Sorbent for VAs in Solid Fraction

Three different extraction solutions were investigated to extract five combined antibiotics from the solid fraction of swine manure (Table 3). There is a significant difference (*p* < 0.05) among the three extraction solution efficiencies. The results show that the T3 method, with an average recovery of 97.36%, depicted a better recovery compared to T1 (24.4%) and T2 (67.67%). Both T2 and T3 are acceptable compared to the literature, due to both having a Na_2_EDTA–McIlvaine buffer, which makes the system operate smoothly and gives a stable recovery. Li et al. [7] mentioned that using the same sorbent of the analytical separation column is considered an advantage and shows a good result. In addition, the targeted VAs in this study are known for their high potential separation performance through C18 column. Karci and Balcioglu [16] reported that 67% was an average recovery of eight VAs from manure using the T3 method. Moreover, an average recovery of five TCs compounds from honey was up to 95.2%, using T3 methods at pH 4.0 using on-line SPE as reported by [7]. Thus, the result showed a higher recovery with the optimized mobile phase than the literature. This could be attributed to the use of an on-line SPE technique combined with UHPLC–DAD.

### 2.3. The Suggested Method

**Figure 3:**
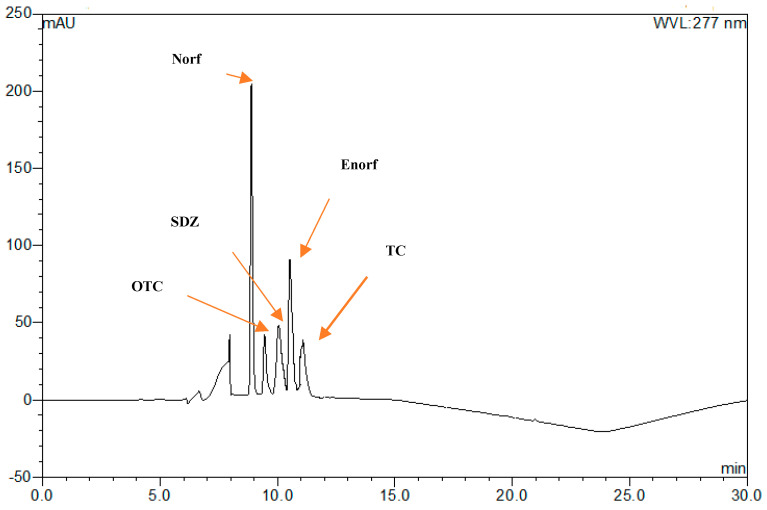
Chromatogram of five VAs (10 µg/mL) in swine manure; retention time = 8.7, 9.14, 9.8, 10.3, and 10.9 minutes, respectively, of Norf, OTC, SDZ, Enorf, and TC at wavelength of 277 nm.

From the aforementioned discussion, it could be suggested that a UHPLC method with diode array detector (DAD) could be combined with on-line SPE and set at wavelengths of 270 and 277 nm for determining five spiked VAs (TC, OTC, Norf, SDZ, and Enorf) from swine manure. The suggested method involves the selection of a suitable mobile phase that contains a mixture of ACN/0.1 M oxalic acid in a gradient elution mode (pH at 3.0), followed by a pre-treatment method for solid fraction samples using 4 mL of an extraction solution containing a mixture between McIlvaine buffer, 0.1 M Na2EDTA solution, and MeOH at a ratio of 25:25:50 (*v/v/v*). The separation step in the suggested method was achieved using a C18 reverse-phase column (AcclaimTM 120, 4.5 × 250 mm, 5 µm particle size), with a column temperature of 30 °C, 35 min as a total run time and injection volume of 50 µL, and an operation flow rate of 0.8 mL min^−1^. By applying the suggested method to blank and spiked samples, optimum chromatograms were obtained (Figure 3), indicating that extraction, clean up, and separation steps are satisfactory to remove the interference of endogenous compounds. However, because the described method has been applied to swine manure that is not treated with the selected antibacterial, investigating this method in swine manure containing excreted antibiotics from animals could give different results. Therefore, the validation of the suggested method was assessed and is stated in the following sections.

### 2.4. Method Validation

The suggested method was validated by evaluating the following parameters: specificity, calibration curve, sensitivity, and within-day and between-day precisions.

#### 2.4.1. Linearity of the Suggested Method

The study was extended to assay the validation of the suggested method, where the obtained result regarding the linearity is shown in Table 4. The linearity was evaluated by generating a calibration curve of 1 to 100 µg mL^−1^ for each compound mixed together at six points in triplicate. Obviously, the DAD response was found to be linear and highly correlated with the injected amount of combined VAs in both the liquid and solid fraction, where the calculated coefficient (R^2^) ranged from 0.995 to 0.999 in the liquid and from 0.991 to 0.999 in the solid fraction. Moreover, the sensitivity (the change in analytical signal units per µm VAs) of the suggested method found to be high, which is usually represented by the slope of the calibration curve [2]. The suggested model linearity of TC, OTC, Norf, SDZ, and Enorf for liquid and solid fractions is shown in Table 4.

#### 2.4.2. Limit of Detection (LOD) and Limit of Quantification (LOQ)

The limit of detection (LOD) is defined as the quantity yielding a detector response approximately equal to thrice the background noise and calculated by the standard deviation of the response (σ) and the slope of the calibration curves [5,14,15]. The limit of quantitation (LOQ) is the lowest amount that can be analyzed within acceptable precision and accuracy at a signal to noise ratio of 10 [19]. In this study, LOD and LOQ were measured, and the obtained data are shown in Table 5. It was observed that the LOD of the suggested method ranged from 0.1 to 0.42 µg mL^−1^ in the liquid fraction and from 0.032 to 0.58 µg g^−1^ dw in the solid fraction, while LOQ values ranged from 0.32 to 1.27 µg mL^−1^ in the liquid fraction and from 0.096 to 1.77 µg g^−1^ dw in the solid fraction.

#### 2.4.3. Precision and Accuracy

Precision is a measure of the results’ variability from the system; commonly, it is described by the within-day and between-day relative standard deviation (RSD%) of a set of replicated results [2]. As shown in Table 4, the within-day precision was calculated over a continuous 4 days by analyzing spiked samples in both matrix fractions, where RSD (%) ranged from 0.29 to 17.85% in the liquid fraction and from 0.44 to 19.48% in the solid fraction, while the between-day precision was found to be in the range of 0.15 to 12.65% in the liquid and 0.44 to 19.8% in the solid fraction. The precision values are in accordance with the European Union regulation 2002/657/EC [20].

## 3. Materials and Methods

### 3.1. Reagents and Materials

**Figure 4:**
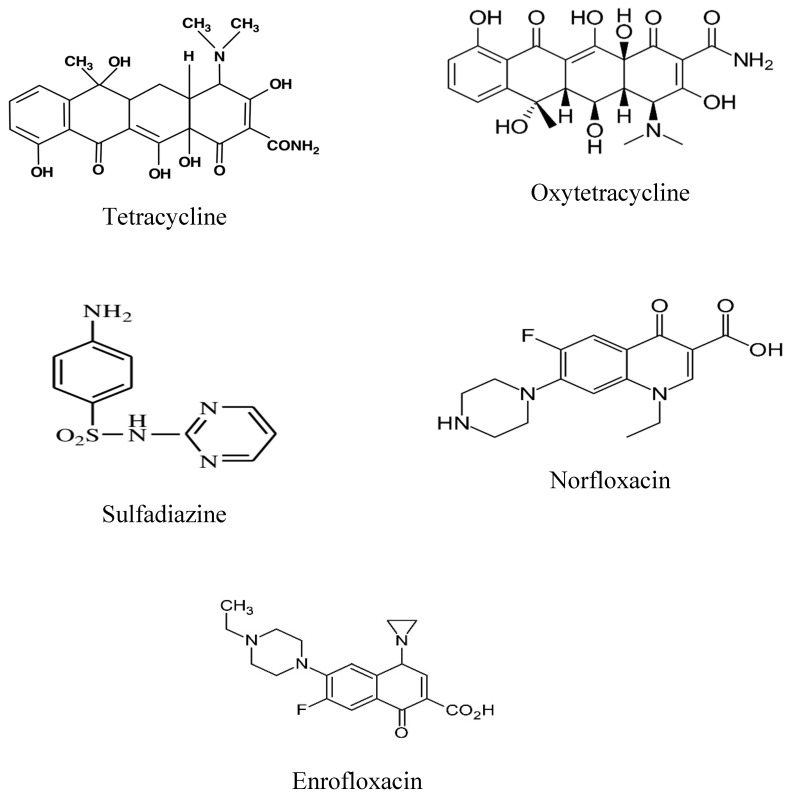
Chemical structure of Tetracycline, Oxytetracycline, Sulfadiazine, Norfloxacin, and Enrofloxacin.

Standards of five veterinary antibiotics (VAs) (Figure 3) (Tetracycline (TC, 90%), Oxytetracycline (OTC, 98%), Sulfadiazine (SDZ, 98%), Norfloxacin (Norf, 98%), Enrofloxacin (Enorf, 98%)) were purchased from Sigma-Aldrich (St. Louis, MO, US) (CAS Reg. No. 60-54-8 for TC, CAS Reg. No. 6153-64-6 for OTC, CAS Reg. No. 68-35-9 for SDZ, CAS Reg. No. 70458-96-7 for Norf, and CAS Reg. No. 93106-60-6 for Enorf) (the structures are presented in Figure 4). Acetonitrile (ACN) and methanol (MeOH) of HPLC grade were purchased from Sigma-Aldrich. Oxalic acid, disodium ethylene–diaminetetraacetat (Na_2_EDTA), disodium hydrogen phosphate (Na_2_HPO_4_), and sodium dihydrogen phosphate (NaH_2_PO_4_) of analytical-reagent grade were obtained from Beijing Chemicals Company (Beijing, China). Deionized water (Milli-QMillipore, Bedford, MA, USA) was used in the study.

### 3.2. Apparatus

An Ultra-High-Performance-Liquid Chromatography (UHPLC) system–Ultimate 3000 Diod Array Detector (DAD) using a Thermo Scientific Dionex Ultimate 3000 system (Thermo Scientific Fisher, DIONEX, Sunnyvale, CA, US) was used, coupled with a thermostatic auto-sampler and an on-line SPE system (Dionex IonPacTM NG1, Guard × 35 mm, CA 94085, US). A C18 reverse-phase column (AcclaimTM 120, 4.5 × 250 mm, 5 µm particle size, Thermo fisher, Sunnyvale, CA, US) was used. The pH meter, ultrasonic bath (Buhler, Leinfelden-Echterdingen, Germany), and freeze-dried machine were also used for sample preparation.

### 3.3. Standard Solutions

Stock standard solutions of each compound of TC, OTC, SDZ, Norf, and Enorf were prepared by dissolving 100 mg of the compound in 100 mL of methanol to obtain a final concentration of 1.0 mg mL^−1^. Stock standard solutions were put in amber glass to prevent photo-degradation and stored at −20 °C and were stable for at least 4 weeks [2,3,5,6,7]. A working mixed-standard solution (100 μg mL^−1^) was diluted using the mobile phase to give a series of dilutions (100, 50, 20, 10, 1, 0.5 and 0.2 µg mL^−1^), and it was stable for at least one week when stored at 4 ℃ in a refrigerator.

### 3.4. Sample Preparation

**Figure 5:**
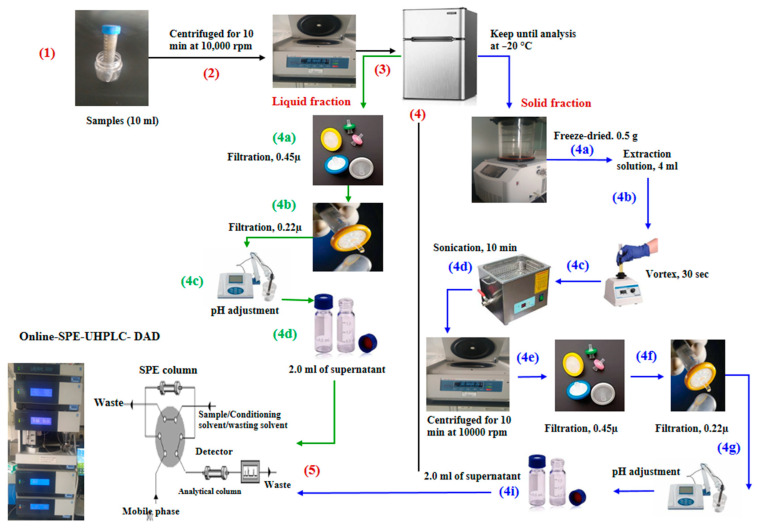
Scheme for the determination of targeted antibiotic residues in swine manure.

Liquid samples: swine liquid manure (5% total solids (TS)) samples were centrifuged at 10,000 rpm for 10 min at 4 °C with a high-speed refrigerated centrifuge (TGL-16 M, Shanghai, China), then the supernatant was filtered through a 0.45 µ filter, and then a 0.22 µ filter. Then samples pH was adjusted to 3.0 by adding H_3_PO_4_ before UHPLC injection step (Figure 5).

Solid samples: 0.5 g of freeze-dried and ground samples were measured and spiked with standard solutions as reported by Marti et al. [21]. Solid samples were extracted by passing through three different solid extraction solutions, where samples were transferred into a centrifuge tube and 4 mL of extraction buffer was added as follows:

i. (T1): a mixture (50:50 *v/v*) of MeOH/ACN and pH adjusted to 3.0;
ii. (T2): saturated aqueous Na_2_EDTA, water, and Na_2_EDTA–McIlvaine buffer (pH 4.0) as developed by Li et al. [7];
iii. (T3): a mixture between a McIlvaine buffer (0.1 M Na_2_EDTA) solution, and MeOH at a ratio of 25:25:50 (based on the volume *v/v/v*), and pH was adjusted to 7.2 by adding 6N NaOH solution. The McIlvaine buffer was prepared by mixing 0.2 M citric acid and 0.4 M Na_2_HPO_4_ solutions at a ratio of (90:60) (based on the volume *v/v*)) as reported by Karci and Balcioglu [16]. The samples’ pH was adjusted to 3.0 before HPLC injection.

Generally, after adding extraction solution, the samples were vortexed for 30 s and then sonicated using an ultrasonic bath (Buhler, Leinfelden-Echterdingen, Germany) for 10 min, then centrifuged at 10,000 rpm for 10 min at 4 °C. The supernatant was filtered through a 0.45 µm filter, and then a 0.22 µm filter. The pH was adjusted to 3.0 by adding H_3_PO_4_.

### 3.5. Cleaning Up

The supernatant was filtered and loaded onto an on-line SPE in an HPLC machine directly.

### 3.6. Chromatographic Separation

Two different mobile phases were finally used after several trials to obtain the best mobile phase mixture for the targeted VAs detection. The first mobile phase consisted of methanol (A), acetonitrile (B), and 0.05 M oxalic acid, as recommended by Abbasi et al. [22], while the second mobile phase was acetonitrile (B), 0.01M oxalic acid (C), and methanol (A) for washing only (Table 2). The prepared mobile phase was filtered through a 0.45 µ filter and then degassed through sonication for 10 min before application. Detection was carried out at 260 nm for TC, 270 nm for Norf and Enorf, and 277 nm for SDZ and OTC. The on-line SPE system was used. A C18 reverse-phase column was used at 30 °C with 35 min as a total run time and an injection volume of 50 µL, with a flow rate of 0.8 mL min^−1^. The software used for control and data acquisition was Dionex™ Chromeleon™ 6.8 (Thermo Fisher Scientific, Sunnyvale, CA, US). External calibration curves were constructed by preparing the standard solutions at six known concentrations (0.5–100 μg mL^−1^ for all VAs). The analytes’ concentration in the sample matrices was determined using peak areas that corresponded to the unknown concentrations in the calibration curve.

### 3.7. Assay Validation

In order to confirm the suitability of the method, it was validated for specificity, linearity, precision, accuracy, limit of quantification (LOQ), limit of detection (LOD), and stability.

#### 3.7.1. Specificity, Linearity, Limit of Detection (LOD), and Limit of Quantification (LOQ)

The validation of specificity, linearity, limit of detection (LOD), limit of quantification (LOQ), recovery yield, and precision for the method was determined. A selected number of swine animals from the National Changping Integrated Agricultural Engineering Technology Center, Livestock and Poultry Branch, Beijing, China were non-medicated. Pig manure was sampled directly from the central pit located at the College of Engineering, China Agricultural University, Beijing, China, sealed well, and stored in a cold, dark room at 4 °C until utilization. Blank samples and spiked samples at 100, 50, 20, 10, 1, 0.5, and 0.2 µg mL^−1^ for both liquid and solid fractions were analyzed on different days, covering all operation conditions. The sample preparation and chromatographic conditions were optimized to guarantee that there were no matrix effects within the retention time of the tested compounds. The response for each antibiotic detected with the HPLC methods was evaluated for linearity, and the limits of detection and quantification for the instrument (LOD and LOQ) were determined, using calibration curves containing six concentration levels (0.5, 1, 10, 20, 50, and 100 mg/L). LOD and LOQ were determined using the standard deviation of the response (σ) and the slope of the calibration curves (S) [23] as follows:

LOD = (3.3 σ)/S(1)

LOQ = (10 σ)/S(2)

#### 3.7.2. Precision

Precision was evaluated by measuring intra-and inter-assay relative RSD%. The intra-assay (within-day) precision was performed by measuring targeted antibiotics in one day. The inter-assay (between-day) precision was determined by analyzing each calibration sample once for 4 consecutive days. Within-day assay and between-day assay precision were expressed as the percentage relative standard deviation (RSD).

#### 3.7.3. Accuracy

Accuracy was expressed as the percentage recovery and calculated as the measured/theoretical value × 100. VAs’ recovery was tested in triplicate for five concentrations (100, 50, 20, 10, and 1 µg mL^−1^).

Recovery (%) = (C1 − C2)/C3 × 100

In which C1 = concentration of analyte in the fortified sample, C2 = concentration of analyte in the non-fortified sample, and C3 = concentration of analyte added to the fortified sample.

## 4. Conclusions

A convenient analytical method using the Ultra-High-Performance Liquid Chromatography method with diode array detection (UHPLC–DAD) coupled with on-line SPE was developed and validated to determine five different veterinary antibiotics (VAs) residues simultaneously in swine manure. Among a wide range of wavelengths, 270 and 277 nm resulted in high peaks. The mobile phase with pH 1.5 performed well; however, the mobile phase (2) with pH 3.0 was recommended due to HPLC stability and performance. Moreover, an optimized solid extraction buffer solution (T3) of VAs from swine manure showed a high extraction efficiency of over 97%. The LOD and LOQ values paired with this method were higher in the liquid fraction than in the solid fraction. The developed method was selective, robust, simple, fast, and inexpensive, and it could be a methodological tool for laboratories dedicated to analyzing emerging pollutants that do not have mass spectrometry detectors.

## Figures and Tables

**Table 1:** The maximum UV-detection wavelengths and retention time of the five drugs.

Antibiotic	Wavelength λ (nm)	Typical Retention Time (min)
OTC	270	9.14
SDZ	277	9.9
Norf	270	8.7
Enorf	277	10.2
TC	270	10.9

**Table 2:** Instrument conditions of UHPLC combined with on-line SPE for analyzing five VAs.

Mobile Phase		Methanol (A) Acetonitrile (B)							
		0.05 and 0.01 M Oxalic Acid in Highly Purified Distilled Water (C)							
Chromatography Condition									
HPLC					On-Line SPE				
Time (min)	Flow Rate (mL∙min^−1^)	A (%)	B (%)	C (%)	Time (min)	Flow Rate (mL∙min^−1^)	A (%)	B (%)	C (%)
0	0.8	0	40	60	0	0.8	0	5	95
10		0	40	60	15		0	5	95
20		0	60	40	20		0	80	20
25		0	40	60	25		0	5	95
30		0	40	60	30		0	5	95
35		100	0	0	35		100	0	0

**Table 3:** Comparison between three different solid extraction methods of 5 combined antibiotics recovery from swine manure.

Solid Extraction Solution	Recovery (%)				
	TC	OTC	SDZ	Enorf	Norf
T1	84.5%	2.85%	11.37%	1.43%	21.87%
T2	77.2%	67.15%	80.63%	62.11%	51.28%
T3	95.58%	84.58%	113.2%	99.73%	93.71%

**Table 4:** Linearity of the suggested method.

Antibiotics	Slope		Intercept		R^2^	
	Liquid	Solid	Liquid	Solid	Liquid	Solid
TC	1.07	1.514	−0.51	−6.62	0.995	0.994
OTC	0.795	0.825	0.05	5.486	0.997	0.992
Norf	2.34	2.85	3.62	5.27	0.999	0.994
SDZ	1.43	1.62	0.19	5.68	0.999	0.991
Enorf	2.17	3.17	1.73	2.18	0.999	0.999

**Table 5:** Limits of detection (3σ), limits of quantification (10σ), standard added recoveries, and relative standard deviations (RSDs, *n* = 6) for the developed method.

Compound	Spiked Level (µg·g^−1^)	LOD		LOQ		*Within-Day RSD (%)*		*Between-Day RSD (%)*	
		Liquid (µg mL^−1^)	Solid (µg·g^−1^)	Liquid (µg mL^−1^)	Solid (µg·g^−1^)	Liquid	Solid	Liquid	Solid
TC	1	0.15	0.29	0.47	0.89	11.27	1.63	9.20	10.58
	10					6.97	4.59	9.02	19.48
	20					8.24	16.94	4.25	9.01
	50					16.9	15.17	3.40	2.26
	100					16.58	14.87	5.87	0.82
OTC	1	0.1	0.58	0.32	1.77	15.52	7.14	6.57	3.65
	10					11.00	17.3	5.24	10.51
	20					1.59	13.67	0.8	12.92
	50					10.87	12.69	11.34	17.45
	100					13.78	7.49	8.21	3.67
Norf	1	0.42	0.032	1.27	0.096	17.78	9.94	8.39	5.56
	10					9.28	13.17	2.81	9.17
	20					4.68	10.32	2.30	9.48
	50					5.5	17.22	4.80	18.06
	100					17.85	10.69	12.65	5.15
SDZ	1	0.34	0.13	1.04	0.39	1.36	8.68	4.47	10.65
	10					5.61	13.73	8.21	0.8
	20					2.15	10.79	1.09	17.28
	50					15.52	1.59	2.86	7.22
	100					9.16	19.8	0.68	4.48
Enorf	1	0.26	0.05	0.79	0.16	7.03	16.93	2.13	12.02
	10					5.37	19.07	6.08	0.44
	20					0.29	6.02	0.15	3.08
	50					12.69	0.87	2.74	10.23
	100					4.32	12.15	3.43	16.24

## Data Availability

Data and all relevant information are available from the authors via email at: jianbinguo@cau.edu.cn (Jianbin Guo) and saadga22@gmail.com (M.S. Gaballah).
